# Admission rates in emergency departments in Geneva during tennis broadcasting: a retrospective study

**DOI:** 10.1186/s12873-018-0209-y

**Published:** 2018-12-13

**Authors:** Jorge César Correia, Olivia Braillard, Christophe Combescure, Eric Gerstel, Hervé Spechbach

**Affiliations:** 1 Division of Tropical and Humanitarian Medicine, Department of Community Medicine, Primary and Emergency Care, Geneva University Hospitals and University of Geneva, 1205 Geneva, Switzerland; 2 Division of Primary Care, Department of Community Medicine, Primary and Emergency Care, Geneva University Hospitals, 1205 Geneva, Switzerland; 3 Division of Clinical Epidemiology, Department of Health and Community Medicine, Geneva University Hospitals, 1205 Geneva, Switzerland; 4 Hirslanden Clinique La Colline, 1206 Geneva, Switzerland

**Keywords:** Emergency, Attendance rates, Sports tournament, Tennis, Workload

## Abstract

**Background:**

Literature provides mixed results regarding the influence of large-scale sporting events on emergency department attendance. To contribute to the research on the subject, we sought to evaluate whether the broadcasting of major tennis tournaments, one of the most popular sports in Switzerland, has an impact on patient admission rates in emergency units in Geneva including 1) type of match 2) the role of a Swiss player, 3) degree of triage, 4) reason of attendance and 5) age of patients.

**Methods:**

Admission rates between periods with tennis matches regarding the semi-finals and finals of 3 major tennis tournaments were compared to equivalent periods without matches from May 2013 to August 2017. Patient admission data was retrieved retrospectively from administrative databases of two Outpatient Emergency Units in Geneva. Patients’ admission rates in periods with and without a tennis match were investigated using a negative binomial regression model with mixed effects.

**Results:**

We observed a statistically significant decrease (− 10%, 95% CI -17 to − 2, *p* = 0.015) in admission rates in periods with a tennis match compared to periods without a tennis match, more pronounced during finals (− 15%, 95% CI -26 to − 3, *p* = 0.017) than during semi-finals (− 7%, − 16 to 2, *p* = 0.13). In addition, this effect was more pronounced in patients aged between 26 to 64 years of age, a category representing professionnally active people. No modification in the admission rates was detected in the hours preceding and following the matches, nor in type of consultations (traumatology vs non traumatology related admissions).

**Conclusion:**

Although modest, the results support the hypothesis that the broadcasting of large-scale sporting events such as tennis matches decreases admission rates in emergency units. Further research is required to explore for a potential causal relationship.

**Electronic supplementary material:**

The online version of this article (10.1186/s12873-018-0209-y) contains supplementary material, which is available to authorized users.

## Background

Emergency Departments (EDs) play an increasingly important role in the care of the population. With demographic changes, increasing life expectancy and greater personal and clinical expectations, attendance rates in EDs have been steadily increasing in Switzerland and worldwide [1–4]. This leads to several challenges such as overcrowding that affects patient care, as well as a worsening of health care providers work related conditions [5–9].

A key strategy is the ability to predict daily attendances in EDs in order to facilitate adequate allocation of resources that can be dynamically tailored to the populations health needs [10–20]. For example, one study proposed a multiple linear regression model based on calendar variables that predicted patient volumes which allowed to optimize staffing patterns. They report that the number of patient complaints dropped by 30% and the number of patients who left without being seen decreased by 18.5% [21].

Another parameter that is being investigated is the effect of large-scale sporting events. However the literature on the subject provides mixed results. While several studies found a statistically significant decrease in attendances during such events [22–31], others have not [32–37].

To contribute to the research on the subject, we decided to evaluate the impact of sports broadcasting on ED attendance in Geneva, Switzerland. We focused on tennis, as it is one of the favorite and most watched sports in the country [38], illustrated by the consistent increase of Swiss TV audience ratings during tennis matches. For the match point in the 2017 final of the Australia Open where Swiss tennis champion Roger Federer claimed victory over Rafael Nadal, 74.4% of the television screens in French-speaking Switzerland were connected to RTS2, the main sports broadcasting channel [39]. Due to this popularity, broadcasting of a tennis match, especially finals and semi-finals is susceptible to influence the attendance.

The aim of our study was to investigate if tennis broadcasting has an effect on ED admission rates. In addition, we investigate the potential effect of 1) type of match 2) the role of a swiss player, 3) degree of triage, 4) reason of attendance and 5) age of patients. Our hypothesis was that broadcasting of major tennis events does affect ED attendance.’

## Methods

### Setting

Data on EDs attendance was collected from two Outpatient Emergency Units, one public and one private, located in the center of Geneva, purposely chosen to ensure equal representation of Geneva’s demographic and socio-economic population.

- Geneva University Hospital’s Outpatient Emergency Unit (HUG): a public university outpatient emergency unit, open 24/7 since November 2016. Before then, the unit was open from 8 a.m. to 11 p.m. On average, the number of yearly consultations is approximatively 35,000 per year; patient median age is 45 years with equal female to male representation.
- Hirslanden Clinique La Colline’s Outpatient Emergency Unit (CLC): a private outpatient emergency unit, open every day of the week from 7 a.m. to 11 p.m. On average, the number of yearly consultations is approximatively 13,000; patient median age is 53 years with equal female to male representation.

Both units use the same patient triage process according to the Swiss Emergency Triage Scale (SETS), ranging from 1 (immediate) to four (non-urgent) according to severity [14]. For degree 1, the patient must be seen immediately, degree 2 must be seen in 20 min, degree 3 in 120 min and degree 4, no time specification (elective consultations).

HUG mostly includes degree three emergency consultations (> 80%) and CLC mostly degree three (60%) and degree four (35%).

### Parameters

In order to evaluate a potential effect of tennis matches on patient attendance rates, we decided to focus on the major annual tennis tournaments which offer the most ranking points, highest prize and, most importantly, highest public and media attention: the Grand Slam tournaments, also called the Majors, and the ATP Finals, known as the Masters.

The Grand Slams consist of the Australian Open in mid-January, Roland Garros in May and June, Wimbledon in July, and the US Open in August and September [40]. The ATP Finals is the second highest tier of men’s tennis tournament after the four Grand Slam tournaments and is held in November [41].

Due to the time difference, we decided to exclude from our study the Australia Open and the US open. In order to increase the study sensitivity, we only focused the analyses on the male semi-finals and finals of each tournament as these are the matches with the highest TV ratings.

All the patients admitted in the two EDs between May 2013 and August 2017 were included.

### Data collection

The frequency of admissions in the two EDs during the tennis matches from May 2013 to August 2017 were calculated from administrative databases. According to the Geneva Ethics committee, no permission was required to search these databases.

The durations of tennis matches were recorded to analyze the admission rates expressed in number of admissions per hour. Only admissions between the exact time of the beginning and the end of matches were included in the analysis. For comparative purposes, control periods were selected for each tennis match and the admission rates at emergency units were calculated for each within the time of the corresponding tennis match. To limit the risk of a confounding phenomenon, control periods were matched to the periods with a tennis match for the year, the season (May–June for Roland Garros, July–August for Wimbledon and October–November for ATP World Tour Finals) and the day of the week. Control periods with another international sport event were excluded (UEFA European Championship: from 10th June to 10th July 2016, Olympic Games: from 5th to 21st August 2016, FIFA World cup: 12nd June to 13th July 2014). For instance, the Wimbledon final in 2014 was held on a Sunday (6th of July) at 1:10 pm and finished at 3:10 pm. The control periods were selected as all Sundays in July and August at the same hours as the final match excluding periods with another major sport event. Data on triage and age of admitted patients were also collected as well as the participation of a Swiss player in the tennis matches.

### Power calculation

The number of periods (sample size) was calculated to reach a power of 80% to detect a difference in mean admission rates between periods with and without a tennis match in each emergency unit. The minimal difference in mean admission rates was expressed in effect size, which is the mean difference divided by the common standard deviation of the admission rates over periods. Indeed, the admission rate varies over periods and we aimed to detect a mean difference important relatively to the natural variability of the admission rates. We assumed a type 1 error of 0.05 (two-sided) and that the standard deviation of the admission rates is equal in periods with and without a tennis match. With 20 periods with a tennis match and 100 periods without a tennis match, the power was 80% to detect an effect size of at least 0.74, which is a difference in mean admission rates equal to 0.74 times the common standard deviation of the admission rates. Since this sample size calculation was conducted for each of the two emergency units, and in a conservative way, the final sample size was 40 periods with a tennis match (which is the number of tennis match from May 2013 to August 2017) and 200 periods without a tennis match.

### Data analysis

The association between the admission rates and broadcasting of a tennis match was assessed using a negative binomial regression model with mixed effects. The dependent variable was the number of admissions during a period and the exposure variable was the duration of the tennis match corresponding to the period. The main dependent variable was the event of a tennis match. Adjustment factors were also introduced in the model (year, season and day of week of the periods). The intercept was random and can vary across clusters (periods with a match and matched control periods). To facilitate the interpretation of results, the regression coefficients were exponentiated so that the associations were expressed as ratios of admission rates. *S*andwich variance estimators were used to assess the *s*tandard errors of regression coefficients.

Two additional negative binomial regression models with mixed effects were conducted refining the main independent variable to investigate if the change in admission rates depended on the stage of tennis match (semi-finals, finals) and on the participation of a Swiss player in the tennis matches. Similar analyses were conducted according to age of admitted patients We distinguished admission rates of three different age groups as a proxy of professional activities: 25 years old or less (professionally inactive), 26 to 64 years and patients (professionally active) and 65 years old or more (professionally retired). All statistical tests were two-sided with a significance level of 0.05.

In order to refine our hypothesis, analysis of admission rate by admission categories were conducted. For simplicity purposes, we categorized admission motives in traumatoloy and non-traumatology, and admission emergency rate in degree 4 and other (degree 1, 2 or 3) according to SETS. Incidence rate ratio (IRR) was calculated with a negative binomial regression model with mixed effects.

Data management and descriptive analyses were performed using R Statistical Software version 3.3.1 (Foundation for Statistical Computing, Vienna, Austria) and regression analyses were performed using STATA version 14.0 (STATA Corp., Texas, USA).

## Results

From May 2013 to August 2017, 40 tennis matches during the selected international tennis tournaments were included in the study (Table 1). Of these matches, 40.7% of semi-finals and 46.2% of finals had the presence of a Swiss player.

**Table 1:** Characteristics of tennis matches included in the study from May 2013 to August 2017

	Finals *n* = 13	Semi-finals *n* = 27
Tournament – n (%)		
ATP World Tour Finals – London^a^	3 (23.1%)	8 (29.6%)
Roland Garros^b^	5 (38.5%)	9 (33.3%)
Wimbledon	5 (38.5%)	10 (37.0%)
Match with a Swiss player – n (%)	6 (46.2%)	11 (40.7%)
Duration of match, minutes - mean (sd)	147 (44)	157 (67)

^a^The final of ATP World Tour Finals – London 16 November 2014 was canceled because of the withdrawal of one player

^b^The 3rd June 2016, the two semi-finals of the Roland Garros tournament were simultaneous. One of the semi-finals was excluded from the analyses

The cumulated duration of the tennis matches was 103 h. In these periods, 413 patients were admitted to the EDs at HUG and 206 patients at CLC. On average, 6.8 control periods were matched per period with a tennis match. The number of admissions during these control periods was 3186 at HUG and 1370 at CLC. Admissions are described in Additional file 1: Table S1.

**Fig. 1:**
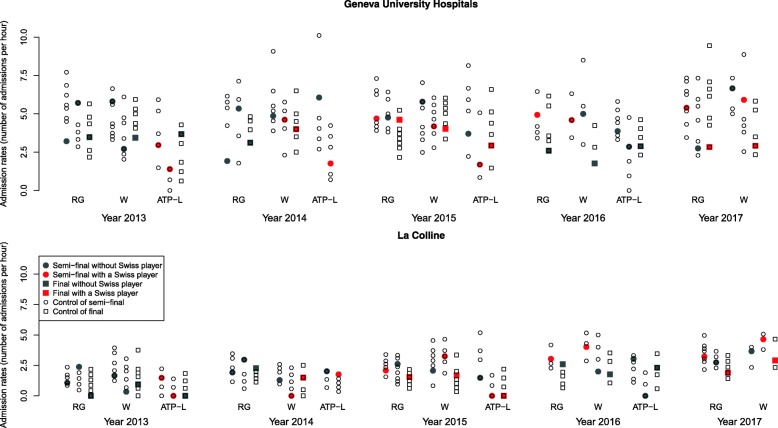
Admission rates (in number of patients per hour) by period and emergency unit. Description: for each match included on the study, the admission rates at emergency unit during the corresponding period (full symbols) and during the matched control periods (empty symbols) are represented. Semi-finals are represented by circles and finals by squares. The year and tournaments (RG: Roland Garros, W: Wimbledon, ATP-L: London) are reported. The color of symbols indicates the participation of a Swiss player to the match (red symbols) or not (grey symbols)

The admission rates are represented in Fig. 1 for each period. The overall admission rates in periods with and without a tennis match are shown in Table 2. The admission rates were globally lower in CLC than in HUG. The unadjusted admission rate ratio was 0.88 (95%CI 0.80 to 0.98, *p* = 0.019) in HUG between periods with tennis matches and periods without tennis matches: The admission rate was 12% lower during tennis broadcasting. In CLC, the change in admission rate had the same direction but was lower: the decrease was 5% and not statistically significant (*p* = 0.34). With adjustment on centers and other factors, the admission rate remained 10% lower in periods with a tennis match compared to periods without a tennis match (Table 3). This reduction was statistically significant (*p* = 0.015). The full results of this model are shown in Additional file 2: Table S2. In an additional model, the decrease in admission rates was 15% during finals and only 7% in semi-finals (Table 3). The decrease in admission rates was similar whatever the participation of a Swiss player to the tennis matches.

**Table 2:** Admission rates at Geneva University Hospitals and at La Colline

	Geneva University Hospitals	La Colline
Periods without tennis match	4.24 (3.79 to 4.75)	1.84 (1.57 to 2.16)
Periods with a tennis match (any match)	3.75 (3.27 to 4.30)	1.74 (1.43 to 2.11)
Periods with a tennis match according to the stage		
Semi-finals	3.93 (3.36 to 4.59)	1.75 (1.42 to 2.14)
Finals	3.33 (2.80 to 3.94)	1.72 (1.18 to 2.32)
Periods with a tennis match according to the participation of a Swiss player		
No participation of a Swiss player	3.68 (3.09 to 4.38)	1.71 (1.35 to 2.15)
Participation of a Swiss player	3.86 (3.32 to 4.47)	1.78 (1.45 to 2.20)

Rates are expressed in number of admitted patients per hour and are reported with 95%confidence intervals in brackets. The admissions rates were assessed using negative binomial regression models with mixed effects

**Table 3:** Associations between the admission rates and the proceeding of a tennis match

	Admission rate ratios	*p*-value
First model		
Periods without tennis match	1 (reference)	
Periods with a tennis match (any match)	0.90 (0.83 to 0.98)	0.015
Second model		
Periods without tennis match	1 (reference)	0.017^a^
Periods with a semi-final	0.93 (0.84 to 1.02)	0.128
Periods with a final	0.85 (0.74 to 0.97)	0.017
Third model		
Periods without tennis match	1 (reference)	0.039^a^
Periods with a tennis match without Swiss player	0.89 (0.79 to 1.00)	0.060
Periods with a tennis match with a Swiss player	0.93 (0.85 to 1.01)	0.080

^a^: Overall *p*-values for testing the equality of admissions rates between the three categories of periods

The associations are expressed in ratios of admission rate during periods with a match compared with periods without tennis match (reference category). A ratio lower than 1 indicates a decrease in the admission rate compared with the reference category, e.g. a ratio of 0.90 in periods with a tennis match means that the admission rate is 10% lower in these periods than in periods without tennis match. All associations were adjusted for centers (HUG/CLC), day of the week, season and year of the tennis match

We investigated possible explanations for these results. First we hypothesized that there might be a shift in patient attendance, or in other words, patients (especially those with non-vital emergencies) are simply occupied watching the tennis matches and will attend the EDs at an ulterior moment. So we calculated the IRR between periods without a match and with a match (any match for primary model and semi-finals or finals for the second model) depending on reason of attendance and emergency degree according to the SETS (Additional file 3: Table S3). The reasons for attendance are shown in Additional file 1: Table S1. Due to incomplete data from CLC, only HUG data is described and analyzed. Thus, results were not adjusted. The proportions of traumatology cases and non-traumatology cases with and without a match were respectively 37% vs 36, and 63% vs 64%. IRR was significantly lower during a match only for non-traumatology cases (IRR 0.87, IC95 0.77–0.98, p 0.024). In the second model, traumatology cases IRR was significantly lower during finals (IRR 0.64, IC95 0.45–0.91, *p* = 0.013).

We then suspected that the tennis matches broadcasting may reduce ‘inappropriate’ consultations defined as patients who attend EDs for conditions that are not true emergencies and don’t require urgent hospital treatment. According to the SETS, this would correspond to degree 4 emergencies which represent less than 10% to the HUG but 30% at the CLC. However, we found that the IRR was significantly lower during a match for degree 1–2-3 (IRR 0.90, IC95 0.82–0.98, *p* = 0.019). This effect was confirmed only in finals (IRR 0.84, IC95 0.72–1.00, *p* = 0.046). No difference was detected in both models for degree 4 cases (Additional file 3: Table S3).

The patients’ admission rates by age are shown in Additional file 4: Table S4. Among patients 25 years old or younger, the decrease in admission rates was observed only for finals (32% lower than in periods without a tennis match) (Table 4). In patients aged from 26 to 64 years, the decrease was similar for semi-finals and finals (15% lower). In contrast, no decrease in admission rates was detected in patients older than 65 years.

**Table 4:** Associations between the admission rates and the proceeding of a tennis match according to the age of admitted patients

	Patients ≤25 years old		Patients from 26 to 64 years old		Patients ≥65 years old	
	Admission rate ratio	p-value	Admission rate ratio	p-value	Admission rate ratio	p-value
First model						
Periods without tennis match	1 (reference)		1 (reference)		1 (reference)	
Periods with a tennis match	1.06 (0.84 to 1.33)	0.632	0.85 (0.77 to 0.94)	0.002	0.98 (0.85 to 1.14)	0.799
Second model						
Periods without tennis match	1 (reference)	< 0.001^a^	1 (reference)	0.007^a^	1 (reference)	0.791^a^
Periods with a semi-final	1.22 (0.93 to 1.60)	0.150	0.85 (0.75 to 0.96)	0.008	1.01 (0.86 to 1.19)	0.920
Periods with a final	0.68 (0.56 to 0.82)	< 0.001	0.85 (0.72 to 1.02)	0.075	0.89 (0.65 to 1.24)	0.499
Third model						
Periods without tennis match	1 (reference)	0.768^a^	1 (reference)	0.004^a^	1 (reference)	0.932^a^
Periods with a tennis match without Swiss player	1.10 (0.85 to 1.44)	0.470	0.80 (0.70 to 0.92)	0.002	1.00 (0.84 to 1.20)	0.980
Periods with a tennis match with a Swiss player	0.99 (0.66 to 1.50)	0.968	0.92 (0.82 to 1.04)	0.196	0.95 (0.73 to 1.24)	0.706

^a^: Overall p-values for testing the equality of admissions rates between the three categories of periods

The associations are expressed in ratios of admission rates during periods with a match compared with periods without tennis match (reference category) and are adjusted for the centers (HUG/CLC)

Table 5 shows the IRR between periods with a final and periods without a match, before, during and after the schedule of the tennis match. The IRR in the 3 h before the match period was significantly higher for traumatology cases (IRR 1.24, IC95 1.01–1.53, *p* = 0.044). Otherwise, no difference of IRR before, during and after the match for all admissions, for non-traumatology cases, by any emergency degree category.

**Table 5:** Incidence rate ratio between periods with a finale and periods without match, before, during and after the schedule of the tennis match

	IRR between periods with a finale and without tennis match		
	Before (3 h)	During match	After (3 h)
All admissions ^a^	0.92 (0.83 to 1.03), *p* = 0.16	0.84 (0.73 to 0.97), *p* = 0.017	0.89 (0.73 to 1.09), *p* = 0.26
Emergency degree ^a^			
1, 2 or 3	0.93 (0.84 to 1.03), *p* = 0.18	0.85 (0.71 to 1.01), *p* = 0.06	0.88 (0.71 to 1.08), *p* = 0.22
4	0.90 (0.56 to 1.44), *p* = 0.66	0.86 (0.60 to 1.23), *p* = 0.40	0.99 (0.65 to 1.50), *p* = 0.95
Reason of attendance^b^			
Traumatology	1.24 (1.01 to 1.53), p = 0.044	no convergence ^c^	0.87 (0.60 to 1.27), *p* = 0.48
Non traumatology	0.90 (0.79 to 1.01), *p* = 0.08	0.87 (0.71 to 1.07), p = 0.18	1.20 (0.93 to 1.55), *p* = 0.15

^a^: Incidence rate ratio adjusted for center

^b^: HUG only

^c^: No convergence of the regression model therefore the IRR could not be estimated

## Discussion

This is the first study of the impact of the broadcasting of a major sporting tournament upon ED attendances in Switzerland. Our results confirm our hypothesis that there is a significant reduction of admission rates during the broadcasting of tennis matches on TV in Switzerland, which concurs with most of the published literature pointing to a decline in the demand for emergency care during major sporting events [22–31]. This effect was more pronounced during finals compared to semi-finals, and for patients in the age group of 26 to 64 years of age (professionally active), without differences between finals and semi-finals for this group. However the difference was not greater during matches with Swiss players. Furthermore, no shift of attendance before or after the matches was observed contrary to the finding in several studies that show an increase in admissions in the hours following these events [23, 28, 31]. Almeida et al. found that the decrease in attendance during such events is especially due to a drop in visits associated with less severe conditions [23] . Our results on the contrary show a decrease in more severe conditions which we found surprising and could not explain. Another aspect we evaluated was the modifications in the type of consultations. Several studies on the subject have demonstrated for example an increase of injuries or intoxication related admission [42–45]. Our results show no such changes. Therefore, no explanation was found to the decrease of attendance observed in our study. This would suggest that certain consultations were just simply “avoided”. Further research is required to explore for a potential causal relationship.

With regards to the lack of effect among the youngest and the eldest patients who consult EDs, we believe that it is related simply to the fact that this population is not as interested in tennis. Indeed, according to a recent survey, the elderly seem more interested in sports such as skiing, ice skating and curling, while the younger generation prefers watching football, ice hockey and rugby [38]. Also, older patients are more often subject to severe acute disease and to multi-morbidity and are therefore more likely to consult in the EDs for a severe medical condition than younger patients.

Regarding the lack of a specific effect of the participation of a Swiss player, we believe that it is related to the fact that people who are fans of Swiss players are just simply fans of tennis in general and enjoy watching other players.

The decrease in attendance observed in our study is quite modest and of too a short duration to justify a modification in the allocation of resources and staffing in the examined EDs. However, it is our opinion that tennis broadcasting can be viewed as another parameter that can be applied to future models used to forecast ED workload. It is important to continue the research on the subject to further study the impact of selected factors on ED admission rates.

The strength of our study is the systematic collection of data regarding admissions to emergency units in both participating centers. Since the time of entries are registered in the databases, we could match periods without tennis broadcasting and with the same timetable than the tennis matches. In addition, by the matching, we controlled for potential confounders such as the day of the week, the season and the year of the tennis matches.

The main limitation of our study is the lack of generalizability of our findings for several reasons. First, the decrease in admission rates is likely related to the television audience during tennis match broadcasting and the higher enthusiasm for tennis observed in Switzerland. The impact of tennis broadcasting on admission rates could be lower, or even null, in countries where tennis is not as popular. Second, only two outpatient emergency units in Geneva participated to the study. It would be of interest to include other outpatient emergency units in Geneva, as well as other Swiss cantons for a broader analysis. Another limitation of our study includes the relatively short period analyzed (4 years), due to the fact that the CLC emergency department was only working at full capacity since 2013. Furthermore, both match and control days used could have been distorted by confounding circumstances such as weather or other environmental conditions, which could have been responsible for variations in the admission numbers. Also, other live and televised events not taken into account could have taken place during control days, thus minimizing the impact described. Finally, the fact we only included outpatient emergency centers in our study might have helped detect the effect on patient attendance in EDs, as patients with more severe disease or injury are more likely to consult EDs when required. Our results might therefore not be generalizable to larger in-patient EDs, like large emergency trauma centers.

## Conclusion

In Switzerland, we observed that the broadcasting of Tennis matches was associated with a decrease in patient admission rates. The underlying reason for this difference is not well understood. Although it may be beneficial to consider the effect of tennis broadcasting on ED service stratification during these periods, the overall effect observed in our study is minimal and will not create a change in future service provision in the selected EDs. Further research is warranted to explore for a potential causal relationship in additional EDs across Switzerland and abroad, in particular at in-patient EDs in order to address the generalizability of the results.

## Additional files

Additional file 1: **Table S1.** Characteristics of admissions to emergency units at Geneva University Hospitals and at La Colline. Description of data: Characteristics of patients admitted to emergency units at Geneva University Hospitals (*n* = 413) and at La Colline (*n* = 206) at the same time as the broadcasting of a tennis match are described by frequencies and percentages. For each participating center, approximately 6 periods without tennis match broadcasting were matched to each periods with a tennis match. Characteristics of patients admitted to emergency units in these periods were also described. (Geneva University Hospitals: *n* = 3186, La Colline: *n* = 1370). (DOCX 14 kb)

Additional file 2: **Table S2.** Association between the admission rate and periods with/without tennis matches, adjusted for the center, the day of the week, the season and the year of the tennis match. The association between the tennis match broadcasting and the admission rate (number of admissions per hour) was investigated by using a multivariable negative binomial regression model with mixed effects. Associations are expressed as ratios of admission rates compared with the reference categories. 95% confidence intervals are reported in brackets. In this model, the intercept was random, for instance the ratio of 0.90 indicated that the admission rate in periods with a tennis match was 0.90 time the admission rate in periods without a tennis match (i.e. 10% lower). The variance of the random effects, 0.03 (95%confidence interval 0.01 to 0.11), indicated that the admission rates varied moderately across periods after adjustment. (DOCX 16 kb)

Additional file 3: **Table S3.** Admission rate ratios (ARR) between periods with and without a match, according to the reason of attendance and emergency degree and adjusted for centers. Description of data: The association between the of a tennis match and the admission rate (number of admissions per hour) was investigated focusing on various types of admissions (traumatology related attendance, non traumatology related attendance, admissions with an emergency degree of 1 to 3 and admissions with an emergency degree of 4). For each type of admission, two negative binomial regression models with mixed effects with adjustment on center were used. In the first model, the “exposure variable” was binary (periods with a tennis versus without a tennis match). In the second model, periods with a tennis match were splitted into two categories (semi-finals and finals). Associations are expressed as ratios of admission rates compared with the reference categories. 95% confidence intervals are reported in brackets. (DOCX 15 kb)

Additional file 4: **Table S4.** Admission rates at Geneva University Hospitals and at La Colline according to the age of admitted patients. The admission rates by center and by category of age of patients assessed using negative binomial regression models with mixed effects. Admission rates are expressed in number of admitted patients per hour and reported with 95% confidence intervals in brackets. (DOCX 12 kb)

## Abbreviations

CLC: Hirslanden Clinique La Colline Outpatient Emergency Unit
EDs: Emergency Departments
HUG: Geneva University Hospital’s Outpatient Emergency Unit
IRR: Incidence rate ratio
SETS: Swiss Emergency Triage Scale
