# Supplementary material for: Admission rates in emergency departments in Geneva during tennis broadcasting: a retrospective study
Source: BMC Emerg Med. 2018 Dec 13;18:56. doi: 10.1186/s12873-018-0209-y (PMC6293595; doi:10.1186/s12873-018-0209-y)
Supplement: Supplementary file 1 — Table S1. Characteristics of admissions to emergency units at Geneva University Hospitals and at La Colline. Description of data: Characteristics of patients admitted to emergency units at Geneva University Hospitals (n = 413) and at La Colline (n = 206) at the same time as the broadcasting of a tennis match are described by frequencies and percentages. For each participating center, approximately 6 periods without tennis match broadcasting were matched to each periods with a tennis match. Characteristics of patients admitted to emergency units in these periods were also described. (Geneva University Hospitals: n = 3186, La Colline: n = 1370). (DOCX 14 kb) [file 12873_2018_209_MOESM1_ESM.docx]

eTable 1: Characteristics of admissions to emergency units at Geneva University Hospitals and at La Colline

|  | Geneva University Hospitals | | La Colline | |
| --- | --- | --- | --- | --- |
|  | Periods with a tennis match | Periods without a tennis match | Periods with a tennis match | Periods without a tennis match |
| Number of admissions | 413 | 3186 | 206 | 1370 |
| Number of periods | 40 | 273 | 40 | 265 |
| Cumulated duration of periods (hours) | 103 | 700 | 103 | 682 |
| Year of admissions, n (%) |  |  |  |  |
| 2013 | 94 (22.8) | 797 (25) | 24 (11.7) | 284 (20.7) |
| 2014 | 70 (16.9) | 544 (17.1) | 33 (16) | 188 (13.7) |
| 2015 | 99 (24) | 846 (26.6) | 43 (20.9) | 373 (27.2) |
| 2016 | 74 (17.9) | 424 (13.3) | 54 (26.2) | 235 (17.2) |
| 2017 | 76 (18.4) | 575 (18) | 52 (25.2) | 290 (21.2) |
| Month of admissions, n (%) |  |  |  |  |
| May-June (Roland Garros) | 171 (41.4) | 1479 (46.4) | 93 (45.1) | 613 (44.7) |
| July-August (Wimbledon) | 183 (44.3) | 1202 (37.7) | 86 (41.7) | 542 (39.6) |
| October-November (ATP World Tour) | 59 (14.3) | 505 (15.9) | 27 (13.1) | 215 (15.7) |
| Age of admitted patients, n (%) |  |  |  |  |
| ≤25 years | 92 (22.3) | 600 (18.8) | 33 (16) | 197 (14.4) |
| 25 to 64 years | 263 (63.7) | 2178 (68.4) | 116 (56.3) | 794 (58) |
| ≥65 years | 58 (14) | 408 (12.8) | 57 (27.7) | 379 (27.7) |
| Emergency degree, n (%) |  |  |  |  |
| 1 | 0 (0) | 6 (0.2) | 2 (1) | 3 (0.2) |
| 2 | 14 (3.4) | 140 (4.4) | 7 (3.4) | 72 (5.4) |
| 3 | 357 (86.4) | 2727 (85.6) | 122 (59.8) | 799 (59.5) |
| 4 | 42 (10.2) | 311 (9.8) | 73 (35.8) | 468 (34.9) |
| missing data | 1 | 2 | 2 | 28 |
| Reason for attendance, n (%) |  |  |  |  |
| Traumatology* | 153 (37.0) | 1148 (36.0) | Nc* | Nc* |
| Non traumatology | 260 (63.0) | 2038 (64.0) | Nc* | Nc* |
| Missing data | 0 | 0 |  |  |

* : not collected (the reason for attendance was not routinely collected in center « La Colline »)
