# Supplementary material for: Admission rates in emergency departments in Geneva during tennis broadcasting: a retrospective study
Source: BMC Emerg Med. 2018 Dec 13;18:56. doi: 10.1186/s12873-018-0209-y (PMC6293595; doi:10.1186/s12873-018-0209-y)
Supplement: Supplementary file 2 — Table S2. Association between the admission rate and periods with/without tennis matches, adjusted for the center, the day of the week, the season and the year of the tennis match. The association between the tennis match broadcasting and the admission rate (number of admissions per hour) was investigated by using a multivariable negative binomial regression model with mixed effects. Associations are expressed as ratios of admission rates compared with the reference categories. 95% confidence intervals are reported in brackets. In this model, the intercept was random, for instance the ratio of 0.90 indicated that the admission rate in periods with a tennis match was 0.90 time the admission rate in periods without a tennis match (i.e. 10% lower). The variance of the random effects, 0.03 (95%confidence interval 0.01 to 0.11), indicated that the admission rates varied moderately across periods after adjustment. (DOCX 16 kb) [file 12873_2018_209_MOESM2_ESM.docx]

eTable 2 : Association between the admission rate and periods without/with tennis match, adjusted for the center, the day of the week, the season and the year of tennis match

|  | Admission rate ratio | p-value |
| --- | --- | --- |
| Periods |  |  |
| Without tennis match | 1 (reference) |  |
| With tennis match | 0.90 (0.83 to 0.98) | 0.015 |
| Center |  |  |
| La Colline | 1 (reference) |  |
| HUG | 2.23 (1.99 to 2.50) | <0.001 |
| Day of the week of the match |  |  |
| Monday | 1 (reference) | <0.001* |
| Friday | 1.22 (0.83 to 1.80) | 0.309 |
| Saturday | 1.13 (0.76 to 1.68) | 0.543 |
| Sunday | 0.97 (0.65 to 1.44) | 0.875 |
| Season of the match |  |  |
| May-June | 1 (reference) | 0.060* |
| July-August | 1.04 (0.96 to 1.13) | 0.343 |
| October-November | 0.72 (0.54 to 0.97) | 0.031 |
| Year of the match |  |  |
| 2013 | 1 (reference) | <0.001* |
| 2014 | 1.15 (0.94 to 1.42) | 0.178 |
| 2015 | 1.26 (1.02 to 1.55) | 0.031 |
| 2016 | 1.31 (1.04 to 1.65) | 0.021 |
| 2017 | 1.48 (1.25 to 1.74) | <0.001 |
| Intercept ** | 1.52 (1.12 to 2.06) | 0.007 |
| logarithm of alpha *** | -4.10 (-4.92 to -3.27) | <0.001 |

* : Overall p-values for testing the equality of admission rates across all categories

** : the intercept is the estimated admission rate in periods without tennis match, at La Colline, a Monday in May-June 2012.

*** : the coefficient alpha is the over-dispersion parameter of the negative binomial regression model.
