# Supplementary material for: Admission rates in emergency departments in Geneva during tennis broadcasting: a retrospective study
Source: BMC Emerg Med. 2018 Dec 13;18:56. doi: 10.1186/s12873-018-0209-y (PMC6293595; doi:10.1186/s12873-018-0209-y)
Supplement: Supplementary file 3 — Table S3. Admission rate ratios (ARR) between periods with and without a match, according to the reason of attendance and emergency degree and adjusted for centers. Description of data: The association between the of a tennis match and the admission rate (number of admissions per hour) was investigated focusing on various types of admissions (traumatology related attendance, non traumatology related attendance, admissions with an emergency degree of 1 to 3 and admissions with an emergency degree of 4). For each type of admission, two negative binomial regression models with mixed effects with adjustment on center were used. In the first model, the “exposure variable” was binary (periods with a tennis versus without a tennis match). In the second model, periods with a tennis match were splitted into two categories (semi-finals and finals). Associations are expressed as ratios of admission rates compared with the reference categories. 95% confidence intervals are reported in brackets. (DOCX 15 kb) [file 12873_2018_209_MOESM3_ESM.docx]

eTable 3: Admission rate ratios (ARR) between periods with and without a match, according to the reason of attendance and emergency degree and adjusted for centers

|  | **Traumatology related attendance** | | | | **Emergency degree** | | | |
| --- | --- | --- | --- | --- | --- | --- | --- | --- |
|  | **Yes** | | **No** | | **1, 2 or 3** | | **4** | |
|  | **ARR** | **p-value** | **ARR** | **p-value** | **ARR** | **p-value** | **ARR** | **p-value** |
| Primary model |  |  |  |  |  |  |  |  |
| No match | 1 (ref) |  | 1 (ref) |  | 1 (ref) |  | 1 (ref) |  |
| Match (any match) | 0.91 (0.74 to 1.11) | 0.36 | 0.87 (0.77 to 0.98) | ***0.024*** | 0.90 (0.82 to 0.98) | 0.019 | 0.95 (0.79 to 1.15) | 0.59 |
| Second model |  |  |  |  |  |  |  |  |
| No match | 1 (ref) | ***0.041*** | 1 (ref) |  | 1 (ref) | ***0.042*** | 1 (ref) | ***0.43*** |
| Semi-finals | 1.03 (0.82 to 1.28) | 0.81 | 0.88 (0.76 to 1.01) | 0.08 | 0.92 (0.83 to 1.02) | 0.12 | 1.01 (0.80 to 1.27) | 0.94 |
| Finals | 0.64 (0.45 to 0.91) | 0.013 | 0.86 (0.71 to 1.04) | 0.12 | 0.84 (0.72 to 1.00) | 0.046 | 0.79 (0.55 to 1.13) | 0.2 |
