# Supplementary material for: Admission rates in emergency departments in Geneva during tennis broadcasting: a retrospective study
Source: BMC Emerg Med. 2018 Dec 13;18:56. doi: 10.1186/s12873-018-0209-y (PMC6293595; doi:10.1186/s12873-018-0209-y)
Supplement: Supplementary file 4 — Table S4. Admission rates at Geneva University Hospitals and at La Colline according to the age of admitted patients. The admission rates by center and by category of age of patients assessed using negative binomial regression models with mixed effects. Admission rates are expressed in number of admitted patients per hour and reported with 95% confidence intervals in brackets. (DOCX 12 kb) [file 12873_2018_209_MOESM4_ESM.docx]

eTable 4 : Admission rates at University Hospitals of Geneva and at La Colline according to the age of admitted patients.

|  | HUG | La Colline |
| --- | --- | --- |
| Admitted patients ≤ 25 years old |  |  |
| Periods without tennis match | 0.85 (0.77 to 0.94) | 0.28 (0.24 to 0.33) |
| Periods with a tennis match (any match) | 0.89 (0.70 to 1.13) | 0.31 (0.21 to 0.47) |
| Admitted patients from 26 to 64 years old |  |  |
| Periods without tennis match | 2.91 (2.60 to 3.26) | 1.08 (0.91 to 1.28) |
| Periods with a tennis match (any match) | 2.39 (2.08 to 2.76) | 0.98 (0.78 to 1.24) |
| Admitted patients ≥ 65 years old |  |  |
| Periods without tennis match | 0.53 (0.78 to 1.23) | 0.51 (0.43 to 0.59) |
| Periods with a tennis match (any match) | 0.52 (0.40 to 0.68) | 0.50 (0.39 to 0.65) |
